# New biogeographical and morphological information on *Physaloptera ngoci* Le-Van-Hoa, 1961 (Nematoda: Physalopteridae) in South-east Asian rodents

**DOI:** 10.1051/parasite/2013023

**Published:** 2013-07-01

**Authors:** Marina Veciana, Kittiponk Chaisiri, Serge Morand, Jordi Miquel, Alexis Ribas

**Affiliations:** 1 Laboratory of Parasitology, Faculty of Pharmacy, University of Barcelona 08028 Barcelona Spain; 2 Department of Helminthology, Faculty of Tropical Medicine, Mahidol University 10400 Bangkok Thailand; 3 Institut des Sciences de l’Evolution, UMR 5554 CNRS-IRDUM2, CC65, Université de Montpellier 2 34095 Montpellier France; 4 Department of Parasitology, Faculty of Veterinary Medicine, Kasetsart University 10900 Bangkok Thailand; 5 Biodiversity Research Institute, Faculty of Biology, University of Barcelona 08028 Barcelona Spain

**Keywords:** Helminths, *Physaloptera ngoci*, Rodents, Thailand, Lao People’s Democratic Republic, Cambodia

## Abstract

During a study of the helminth fauna of 1,643 rodents trapped along the Mekong River (Thailand, Lao People’s Democratic Republic and Cambodia) in 2008–2011, the spirurid nematode *Physaloptera ngoci* Le-Van-Hoa, 1961 was recovered with an overall prevalence of 2.8%. Based on the original description, it was identified in nine of 23 different Murinae host species and is here reported for the first time from these three countries. A scanning electron microscopy study provides additional morphological data.

## Abstract (fr)

Lors de l’étude des helminthes de 1,643 rongeurs, capturés le long du Mékong en Thaïlande, République Démocratique Populaire du Laos et Cambodge entre 2008 et 2011, le nématode Spiruridae *Physaloptera ngoci* a été détecté avec une prévalence globale de 2.8 %. En accord avec la description originale, *P. ngoci* a été identifié chez neuf des 23 espèces de Murinae étudiées et est signalé pour la première fois dans ces trois pays. Une étude en microscopie électronique à balayage apporte des nouvelles données morphologiques.

## Introduction

Unnamed species of *Physaloptera* Rudolphi, 1819 were reported from South-east Asia (SEA) in rodents belonging to the family Muridae on a number of occasions. In West Java (Indonesia), *Physaloptera* sp*.* was recorded in *Maxomys bartelsii* (as *Rattus bartelsii*) and *Niviventer lepturus* (as *Rattus lepturus*) [16]; in the same area, Hasegawa *et al*. [4] found specimens of *Physaloptera* in *Rattus argentiventer*. In Malaysia, immature stages of *Physaloptera* sp*.* were reported in seven different rodent species: *Berylmys bowersi*, *Leopoldamys sabanus*, *Maxomys rajah*, *R. argentiventer* (as *Rattus rattus argentiventer*), *Rattus tiomanicus* (as *Rattus jalorensis* and *Rattus rattus rumpia*), *Sundamys mulleri* (as *Rattus mulleri*) [15] and *Rattus* sp*.* [11]. Unnamed specimens of *Physaloptera* were also reported from Asia outside SEA, namely from Japan in *Apodemus agrarius* and *Rattus rattus* [3] and from India in *R. rattus* (as *Rattus rattus rufescens*) and *Tatera indica* (as *Tatera indica indica*) [10].

*Physaloptera* species were also reported from the SEA region in mammals of the family Sciuridae, specifically *Physaloptera inermis* Linstow, 1906 in *Callosciurus prevostii* (as *Sciurus prevostii*) from Malaysia and *Physaloptera sciuri* Parona, 1898 in *Callosciurus melanogaster* (as *Sciurus melanogaster*) from Indonesia [5, 13]. In the Indian region (close to SEA) *Physaloptera funambuli* Parihar & Nama, 1978 was detected in *Funambulus pennanti* [10, 12].

Bearing the above-mentioned undetermined species in mind, *Physaloptera ngoci* is to date the only known species of this genus in Muridae from SEA. *Physaloptera ngoci* was also described from Vietnam in *Rattus norvegicus* [5] and subsequently recorded in *Mus* sp. and *Rattus* sp. [14].

In the present study, rodents were captured in SEA during 2008–2011 and their helminths, including *P. ngoci*, recovered.

## Materials and methods

**Figure 1.**
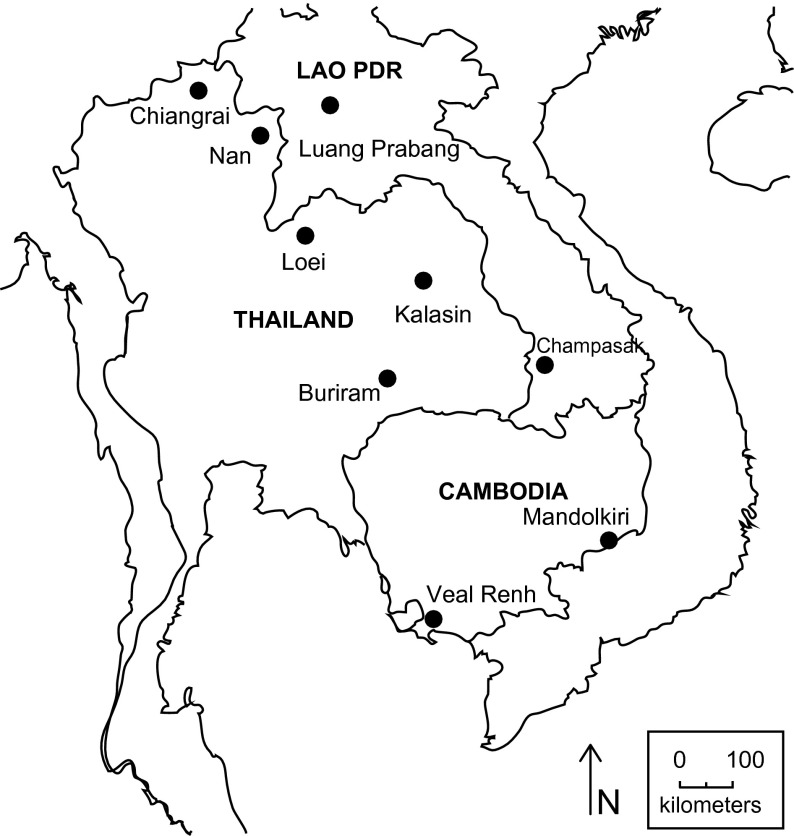
Location of nine CERoPath sampling sites in South-east Asia.

Murid hosts were captured at nine different sampling sites along the Mekong River in Thailand, the Lao People’s Democratic Republic (PDR) and Cambodia (Figure 1). The rodents were trapped with baited traps, either locally made or Sherman. At each sampling locality, ten trap lines (composed of ten traps, placed every five metres) were set over a period of 12 nights. Trap lines were moved every four nights. Complementary trapping was carried out in villages and isolated houses, with five traps per house. Rodents were identified by morphology or using species-specific primers and/or barcoding assignments (details available in the *Barcoding Tool/RodentSEA* section of the CERoPath project website: www.ceropath.org). Rodents were euthanized and dissected following protocols that maximize animal care, the health and safety of field parasitologists and the generation of quality data.

Viscera were examined under a stereomicroscope and helminth parasites were collected and preserved in 70% ethanol. Nematodes were cleared in lactophenol and hand-cut sections of the male anterior end were mounted *en face* for identification. Females were dissected in order to count the number of uteri. Photographs and measurements were taken with a microscope mounted on a Tucsen camera with associated software. Measurements taken from 10 adult individuals of each sex (males from *Bandicota berdmorei*, *Bandicota indica* and *Bandicota savilei* and females from *B. berdmorei*, *B. indica* and *R. argentiventer*) are given in micrometres unless otherwise specified and listed as range and median.

For Scanning Electron Microscopy (SEM) studies, nematodes were fixed in 70% ethanol, critical point-dried, mounted on SEM studs and finally coated with a thin layer of platinum before being examined with a Zeiss DSM 940A Scanning Electron Microscope at an accelerating voltage of 15 kV.

A subset of studied specimens was deposited in the Museu de Ciències Naturals de Barcelona, Catalonia, Spain, with Accession Numbers MZB 2013-0019 and MZB 2013-0020 (two females), and MZB 2013-0021 and MZB 2013-0022 (two males). Murid hosts were identified following the nomenclature and classification in Chaval [1].

## Results and discussion

A total of 1,643 rodents belonging to 23 species were examined and in nine of these species a nematode belonging to the genus *Physaloptera* was isolated from the stomach and identified as *P. ngoci* (details of hosts and prevalence are summarized in Table 1). The general prevalence was 2.8%, the highest prevalence being 40% in *R. argentiventer* (*n* = 10) from Veal Renh, Cambodia. *Physaloptera ngoci* was absent in the following rodents: *B. bowersi* (*n* = 27), *Chiropodomys gliroides* (*n* = 2), *Hapalomys delacouri* (*n* = 1), *Leopoldamys edwardsi* (*n* = 14), *L. neilli* (*n* = 1), *Maxomys surifer* (*n* = 81), *Mus cookii* (*n* = 141), *M. fragilicauda* (*n* = 1), *Mus* sp*.* (*n* = 3), *Niviventer fulvescens* (*n* = 76), *Rattus andamanensis* (*n* = 1), *R. nitidus* (*n* = 14) and *R. norvegicus* (*n* = 17).

**Table 1.** Prevalence (%) of *Physaloptera ngoci* and number of rodents (*n*) examined at study sites in Thailand, Lao People’s Democratic Republic and Cambodia (localities within the same country are pooled).

Hosts	Thailand	Lao	Cambodia	Overall (*n* = 1,643)
*Bandicota indica*	6.2% (*n* = 177)	0% (*n* = 3)	0% (*n* = 1)	6.1% (*n* = 181)
*Bandicota savilei*	13.6% (*n* = 22)	100% (*n* = 1)	8% (*n* = 50)	11.0% (*n* = 73)
*Berylmys berdmorei*	5.3% (*n* = 19)	0% (*n* = 3)	0% (*n* = 6)	3.6% (*n* = 28)
*Mus caroli*	1.3% (*n* = 76)	0% (*n* = 39)	0% (*n* = 1)	0.9% (*n* = 116)
*Mus cervicolor*	0.8% (*n* = 130)	–	–	0.8% (*n* = 130)
*Rattus argentiventer*	0% (*n* = 7)	–	40% (*n* = 10)	23.5% (*n* = 17)
*Rattus exulans*	0.5% (*n* = 214)	0% (*n* = 50)	1.5% (*n* = 67)	0.6% (*n* = 331)
*Rattus sakeratensis*	10.9% (*n =* 92)	10% (*n* = 10)	–	10.8% (*n* = 102)
*Rattus tanezumi*	0% (*n* = 134)	0% (*n =* 90)	11.3% (*n* = 62)	2.5% (*n* = 286)
Total (*n* = 1,643)	2.6% (*n* = 1074)	0.7% (*n* = 287)	5.7% (*n* = 282)	2.8% (*n* = 1643)

The observation of structures in the cephalic region, the distribution of the cephalic papillae, the arrangement and number of male caudal papillae, the size of the male spicules, the number of female uteri, the total length and the distance from the anterior end to the vulva all confirm the identification of this helminth as *P. ngoci*, as per the original description [5].

**Figure 2.**
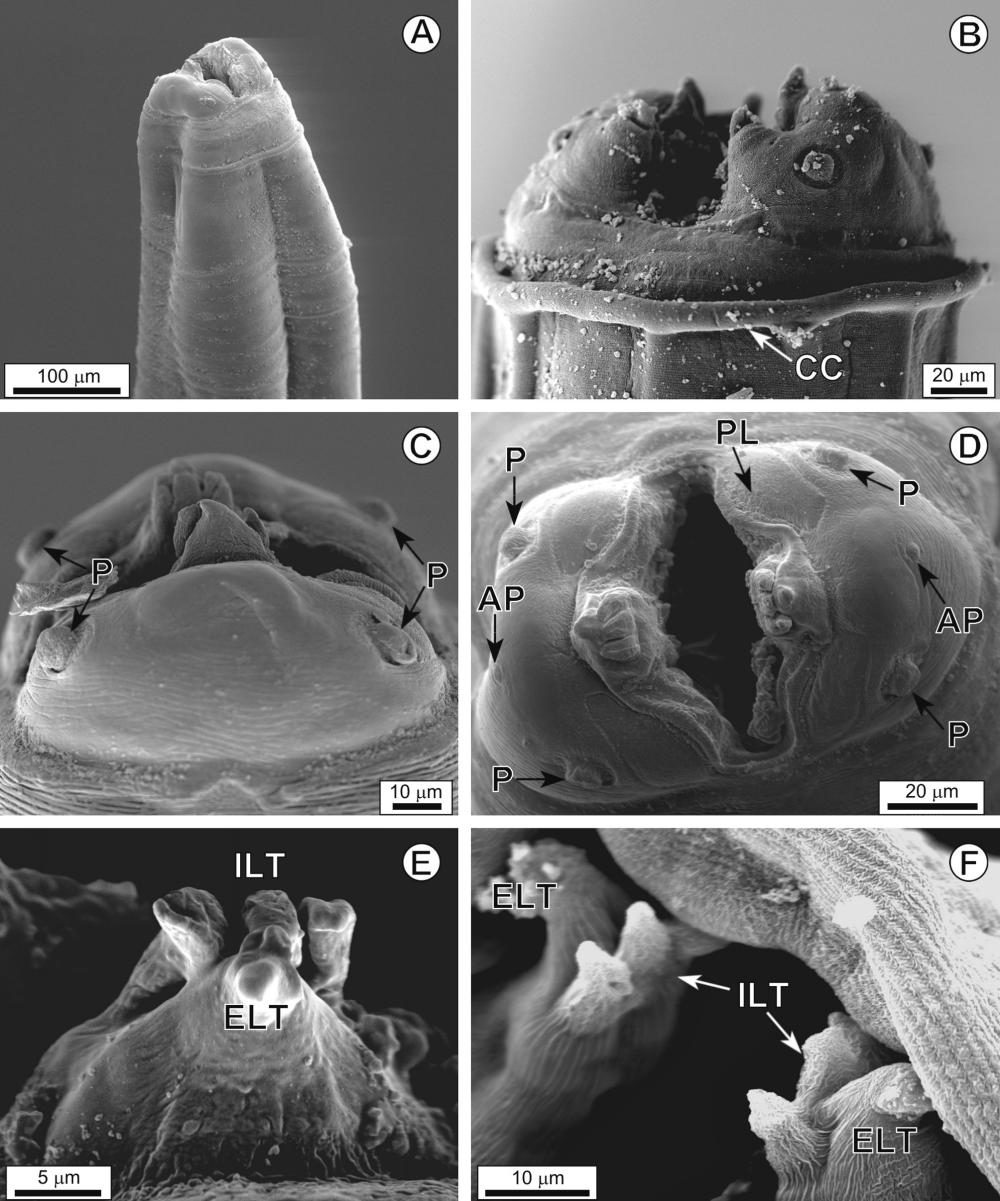
*Physaloptera ngoci*. A, cephalic extremity, general view. B, cephalic extremity, lateral view, cephalic collarette (CC). C, cephalic extremity with detail of papillae (P), lateral view. D, cephalic extremity, apical view. AP, amphids; PL, porus-like region; P, papillae. E, F, detail of externolateral tooth (ELT) and tripartite internolateral tooth (ILT).

**Figure 3.**
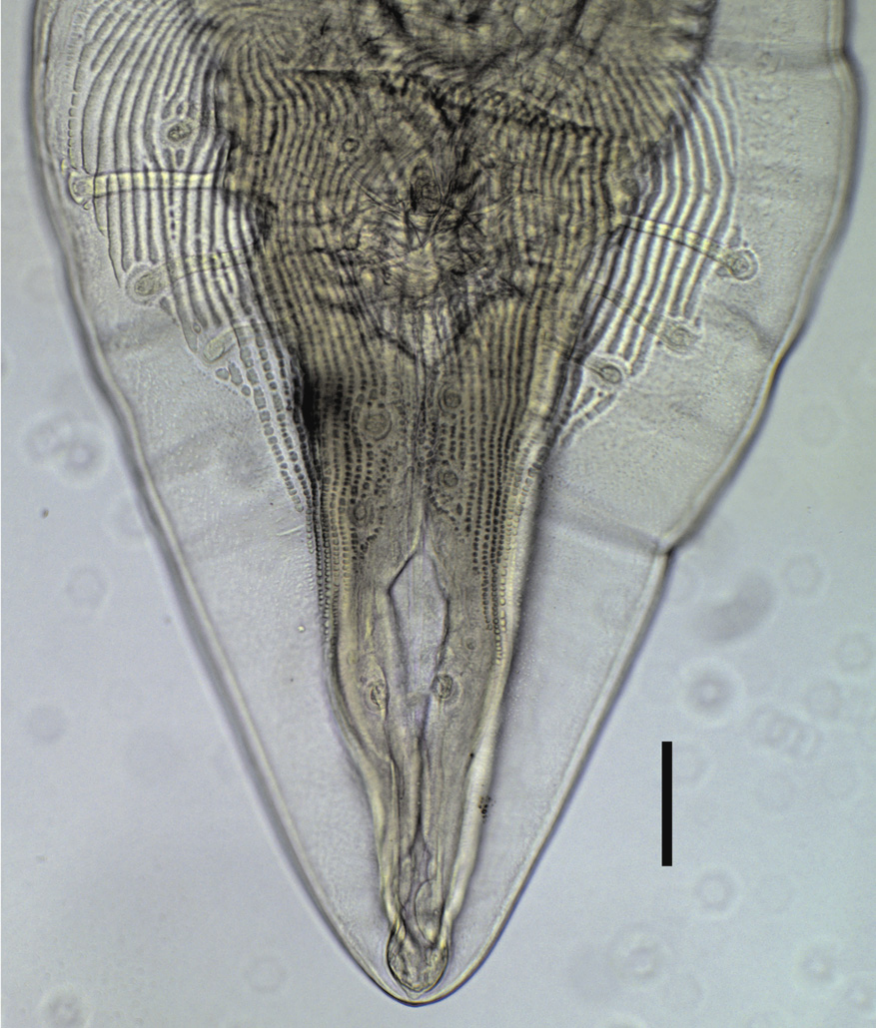
*Physaloptera ngoci* male. Caudal papillae, ventral view. Scale bar: 100 μm.

The cephalic region (see Figure 2A–F) bears two semicircular pseudolips, each bearing a pair of papillae, one amphid, a single externolateral tooth and a trident-like internolateral tooth. The arrangement of the 21 caudal papillae in males is as follows: (i) four pairs of pedunculate papillae (one pair precloacal, one adcloacal and two postcloacal) and three single sessile papillae, distributed as a precloacal pair and a single adcloacal papilla; (ii) two pairs of sessile, immediately postcloacal papillae and (iii) four pairs of sessile papillae located on the tail, distributed in two groups of two pairs, one group towards the middle and the other nearer the end of the tail (Figure 3). Left spicule 370–574 (470) and right spicule 259–354 (296) long. Female total length 17.92–28.74 (21.17) mm. Distance from the anterior end to the vulva 2.06–4.58 (3.48) mm. Vulva in the first third of the body. Four uteri. Eggs 23.6–33.3 (28.1) × 38.4–48.6 (44.5).

The morphology of our samples confirms the identification of this nematode as *P. ngoci* given the similarity of structures and their disposition in the cephalic region, the same number and similar arrangement of male caudal papillae, and the same number of uteri in the females. In addition, our measurements match those provided in the original description: left and right spicule 460 and 300 long, respectively, total length 25 mm in females, distance from the anterior end to the vulva 3.32 mm and egg size 28 × 45 [5].

Of the congeners previously recorded from small mammals in India and the SEA region, all share the presence of four uteri in the females, but *Physaloptera funambuli* differs from *P. gnoci* in the number of male caudal papillae (10 pairs), a much longer left spicule 2.72–2.88 (2.80) mm and a shorter right spicule 0.19–0.20 (0.193) mm [12]. The total female length is greater 27.55–49.20 (35.12) mm [12], as is the distance from the anterior end to the vulva 4.67–9.26 (5.88) mm [12]. Eggs are smaller 20–40 (24) × 20–45 (28) [12].

*Physaloptera inermis* differs from *P. gnoci* in the number of male caudal papillae (eight pairs) and longer spicules 2.37 mm [6] (no differences between right and left spicule specified). The total female length is greater 51 mm [6]. The distance from the anterior end to the vulva is not given in the original description. Egg size is similar 26 × 47 [6].

*Physaloptera sciuri* differs in the number (15) and disposition of male caudal papillae (no sessile precloacal papillae, one unpaired sessile postcloacal papilla and the final pair of postcloacal papillae close to the tail). Spicule lengths are not provided in the original description. The total female length 17–32 mm is similar [13]. The vulva is approximately halfway down the body and we were clearly able to differentiate its position from that in *P. gnoci*, in which it is more anteriorly. The eggs are smaller 16 × 22 [13].

SEM studies are currently available for four species of *Physaloptera*: *Physaloptera sibirica* Petrow & Gorbunow, 1931 in mammals from Spain [9]; *Physaloptera bispiculata* Vaz & Pereira, 1935 in the South American water rat *Nectomys squamipes* from Brazil [8]; *Physaloptera* sp*.* in an anuran from Argentina [2]; and *Physaloptera herthameyerae* Lopes *et al.*, 2009 in a marsupial from Brazil [7]. The present study increases the number of SEM studies in *Physaloptera* to five and is the first such study done on a species from hosts in SEA.

This is the first report of *P. ngoci* from rodents in Cambodia, Lao PDR and Thailand. While its original description was based on specimens from a single host species, the brown rat (*R. norvegicus*) from Vietnam, several new hosts are listed for *P. ngoci* in the present study. Its host spectrum now includes several species of the subfamily Murinae; likewise, its known geographic range is expanded to include SEA. Previous reports of *Physaloptera* spp. in SEA murine rodents can thus probably be assigned to *P. ngoci* given that our data confirm that this nematode is widespread in SEA.
